# Holistic face perception in young and older adults: effects of feedback and attentional demand

**DOI:** 10.3389/fnagi.2014.00291

**Published:** 2014-10-22

**Authors:** Bozana Meinhardt-Injac, Malte Persike, Günter Meinhardt

**Affiliations:** Department of Psychology, Johannes Gutenberg University MainzMainz, Germany

**Keywords:** age-related decline, holistic face perception, composite effect, attentional focus, attentional demand

## Abstract

Evidence exists for age-related decline in face cognition ability. However, the extents to which attentional demand and flexibility to adapt viewing strategies contribute to age-related decline in face cognition tests is poorly understood. Here, we studied holistic face perception in older (age range 65–78 years, mean age 69.9) and young adults (age range 20–32 years, mean age 23.1) using the complete design for a sequential study-test composite face task (Richler et al., 2008b). Attentional demand was varied using trials that required participants to attend to both face halves and to redirect attention to one face half during the test (high attentional demand), and trials that allowed participants to keep a pre-adjusted focus (low attentional demand). We also varied viewing time and provided trial-by-trial feedback or no feedback. We observed strong composite effects, which were larger for the elderly in all conditions, independent of viewing time. Composite effects were smaller for low attentional demand, and larger for high attentional demand. No age-related differences were found in this respect. Feedback also reduced the composite effects in both age groups. Young adults could benefit from feedback in conditions with low and high attentional demands. Older adults performed better with feedback only in trials with low attentional demand. When attentional demand was high, older adults could no longer use the feedback signal, and performed worse with feedback than without. These findings suggest that older adults tend to use a global focus for faces, albeit piecemeal analysis is required for the task, and have difficulties adapting their viewing strategies when task demands are high. These results are consistent with the idea that elderly rely more on holistic strategies as a means to reduce perceptual and cognitive load when processing resources are limited (Konar et al., 2013).

## 1. Introduction

As it is found for other cognitive abilities, face cognition performance also undergoes age-related decline (Bartlett et al., 1989; Crook and Larrabee, 1992; Searcy et al., 1999; Pfutze et al., 2002; Chaby et al., 2003; Hildebrandt et al., 2010; Germine et al., 2011). Clearly, the well-documented decline in memory function with age may, at least partly, underlie the decline observed in face recognition tests (Fulton and Bartlett, 1991), and loss in general perceptual functions (Sekular and Sekular, 2000; Lott et al., 2005), speed limitations (Salthouse, 1996, 2000), and top-down suppression and attentional control (Gazzaley et al., 2005a,b, 2008) may contribute to these effects. Because there are multiple sources of age-related decline it is hard to judge whether impaired performance of the elderly is due to a decline in face-specific mechanisms or to impairment in general cognitive functioning, which is necessarily involved in face cognition tests.

Recent cross-sectional studies (Wilhelm et al., 2010; Hildebrandt et al., 2011) revealed that face cognition ability is predicted by non-facial general ability in memory function, speed, and object cognition with about 50% explained variance. The degree of predictability proved to be relatively stable across young, middle, and late adulthood, indicating no age-related dedifferentiation of face and non-face cognition (Hildebrandt et al., 2011). These findings suggest that the special status of face cognition, as a set of distinct abilities, is preserved in late adulthood.

A key feature that characterizes face cognition as a distinct and highly developed ability is its holistic nature. Processing of face parts is highly sensitive for the facial context such that a change of parts usually changes the overall appearance of a face. Striking demonstrations are the “part-to-whole effect” (Tanaka and Farah, 1993; Tanaka and Sengco, 1997) and the “composite effect” (Young et al., 1987). The part-to-whole effect shows that facial features are more easily identified when they appear in their natural face contexts. The composite face effect shows that upper and lower face halves interact perceptually, and cannot be judged independently. When two composite faces are shown that combine upper and lower face halves from different persons, observers have difficulty matching the identity of only the upper or lower halves (see example in **Figure 3**). Meanwhile, the composite effect is frequently used to assess holistic face processing (for an overview, see Rossion, 2013).

Aging studies have addressed whether age-related decline exists in the ability to apply holistic viewing strategies for faces. Corresponding to Wilhelm et al. (2010) and Hildebrandt et al. (2011), recent studies have corroborated that the integrative nature of face processing is not affected by aging. A study with young and older adults (mean age 68.6) found age-related decline in face identification was reported, but, no decline in the composite effect (Konar et al., 2013). Further, the composite effect predicted face identification performance to the same degree in both age groups, which indicates that the association of holistic face perception and face cognition is maintained at mature ages.

Meinhardt-Injac et al. (2014b) studied how external features modulate the perception of internal features in young and older adults (mean age 70.4), and tested the accuracy of sequentially matching faces by attending either feature class. They found about equally strong holistic effects in both age groups. However, older adults performed better with external features, while the accuracy of assessing the inner face details declined. Daniel and Bentin (2012) recorded the face specific N170 potential and the P300 component to assess global, configural and featural face-processing strategies in younger and older adults (mean age 77.1). They found that older adults processed faces by relying on global features, which shows deficits in tasks that require local configural cues. Taken together, present evidence suggests that the elderly do not suffer from deficits in the ability to process faces holistically, but may have difficulties attending diagnostic facial cues. These findings point to the role of attentional capabilities.

Ample evidence exists that attentional selection of older adults is impaired in tasks with simultaneous presentation of target and non-target stimuli (Quigley et al., 2010; Schmitz et al., 2010). Further, serious age-related deficits have been reported for tasks that require one to change attentional focus during a trial (Georgiou-Karistianis et al., 2006). However, in tests of holistic face processing, researchers have assessed how unattended facial features affect the judgments of attended facial features. Holistic face processing is concluded from the failure to selectively attend to face parts (Richler et al., 2008b). Therefore, the sensitivity of holistic face perception should be controlled in regard to the variation of attentional task demands. If higher attentional demands yield stronger holistic effects for older adults, then their preference for global and holistic viewing strategies would, at least partly, be due to age-related decline in attentional mechanisms (see Discussion).

**Figure 1:**
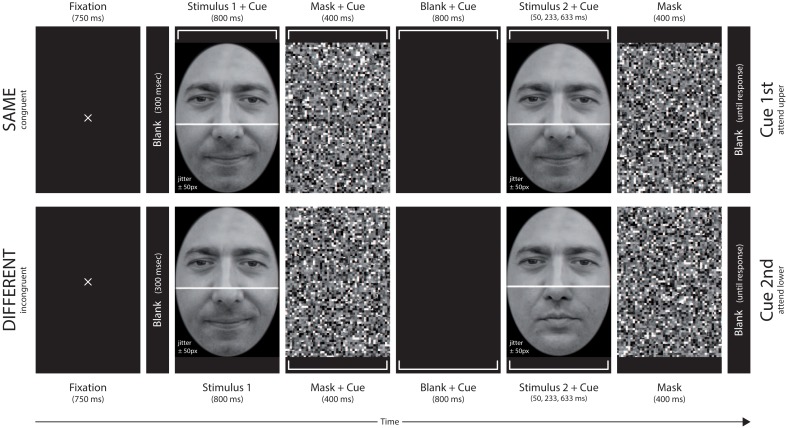
**Examples of a single trial for the *1st cue* (upper row) and the *2nd cue* (lower row) condition**. The white brackets informed the observer whether upper or lower face halves were to be compared. The upper row shows a *same* trial in congruent condition with upper target half and early cue, the lower row a *different* trial in incongruent condition with lower target half and late cue.

The effect of attentional demand can be examined in a sequential study-test composite face task by varying the temporal position of the cue that indicates which of both face halves, the upper or lower, have to be attended (see Figure 1). If the cue comes with the study image (Figure 1, upper panel) the observer can try to attend just the cued half and maintain the attentional focus throughout the trial. If the cue comes after the study image (Figure 1, lower panel) she/he must attend to both halves, and switch attention toward the target test half within the trial.

The effects of the unattended face halves are expected to be stronger in the late cue condition because the whole study face is attended. Both conditions differ in two respects, which are relevant for comparison among age groups. First, the late cue condition requires one to change the attentional focus from the whole study face toward only one half during the test. If age-related differences in attentional control and reallocation of resources modulate performance, the effect of cue position should be different in both age groups. Second, varying the temporal cue position alters not only attentional requirements, but also memory demands. In the late cue condition, features from the upper and lower halves must be encoded and held in memory until the test. If working memory load is crucial for performance in the composite face task, a differential effect of cue position in both age groups should also exist because working memory capacity differs strongly among young and old adults (Brockmole and Logie, 2013). Hence, varying the temporal cue position can reveal whether age-related differences in coping with increased task demands in the composite face task.

A further aspect is the role of cognitive control that may be used to regulate the influence of the unattended on the attended facial features. Meinhardt-Injac et al. (2011) observed that young adults could enhance accuracy in judging the identities of internal features in the presence of incongruent external features by about 10% when trial-by-trial feedback about correctness is provided. This result was stable for exposure durations between 200 and 650 ms, indicating that young adults are able to replace holistic features by piecemeal face processing if they have sufficient temporal resources and the opportunity to adjust their viewing strategy with the help of feedback. For older adults, the role of feedback for optimizing the face viewing strategy, to date, has not been addressed.

The focus of the present study was threefold. First, we remeasured the composite effect for young and older adults because the current state of evidence for maintenance of holistic face perception at mature ages is not yet settled. In recent studies (Boutet and Faubert, 2006; Konar et al., 2013), the composite face effect was examined by comparing aligned and misaligned composite face arrangements, as in the seminal study on the composite effect (Young et al., 1987). However, in the last years, there was progress in the methodological development of the composite face paradigm, leading to a fully balanced and complete design (Gauthier and Bukach, 2007; Cheung et al., 2008). We decided to use this new design because of its methodological advantages (see Methods) to first add results on aging effects with the complete design to the literature. Second, we varied task demands, allowing the observer to select the attentional focus in advance and maintain it throughout the trial, or to force her/him to reallocate attentional resources during a brief time interval. Comparing across age should dismantle age-related capabilities and limitations in coping with higher task demands. Third, we provided trial-by-trial feedback, or not, to reveal whether older adults are able to use higher-level cognitive control to learn and refine their viewing strategies in the same way as young adults.

## 2. Materials and methods

### 2.1. Experimental outline

We used a variety of the sequential composite face tasks (Richler et al., 2008b). In the experimental trials, subjects first fixated on the screen center and then saw a composite study face. The image remained on the screen for 800 ms. After masking with a carefully designed mask pattern (see below), another blank screen interval followed, and then the composite test face was presented for one of three possible presentation times chosen at random. Subjects then decided by button press whether the study and test agreed or disagreed in the face halves that were being attended (upper or lower). In the first cue condition, a large white bracket marking the face half to be attended was shown with the study image. In the second cue condition, the bracket appeared after the study image, together with its subsequent mask (see Figure 1).

Cue position conditions were run in separate experimental blocks because pilot measurements showed that the task was too hard for the elderly if the target cue position was varied randomly interleaved. Each experimental block was run with acoustical trial-by-trial feedback about correctness and without. Three exposure durations were chosen for the test image, one brief timing precluding saccades and serial scans (50 ms), an intermediate timing (233 ms), and a relaxed timing (633 ms) to allow for detailed image scrutiny.

### 2.2. Experimental design

**Figure 2:**
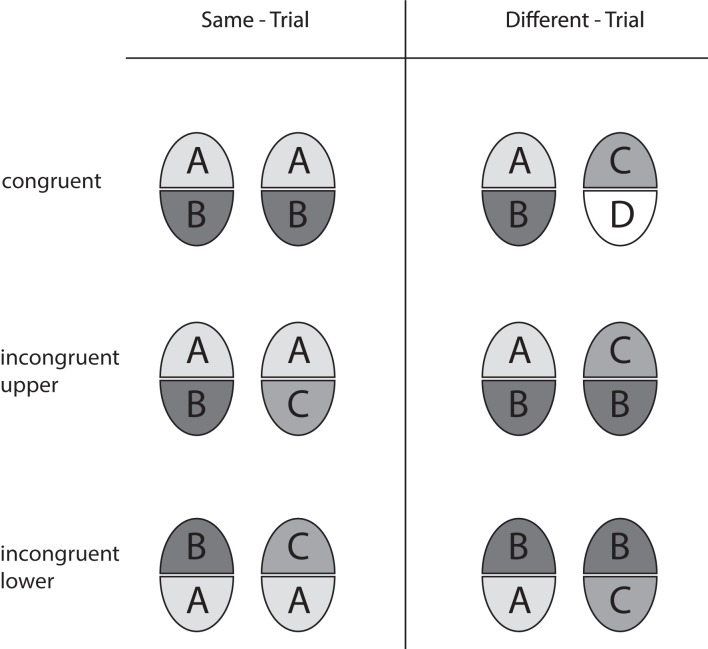
**Overview of the trial types used in the complete design with upper and lower face halves as the targets**. Equal halves of a composite face pair are marked by same letters and same levels of gray.

We employed the “complete design” (CD) of the composite face paradigm (Gauthier and Bukach, 2007; Cheung et al., 2008). In contrast to a former variety (called the “partial design,” PD, by Cheung and colleagues) congruent and incongruent face half pairings are fully balanced in the CD, and performance in terms of accuracy as well as holistic effects are calculated from both response categories in order to avoid confounds with a possible preference (bias) toward either response category. The design is illustrated in Figure 2. Same-trials and different-trials are realized in the *congruent* and the *incongruent* variety. In congruent trials, the non-attended halves agree when the attended halves agree (same-trial), and disagree when the attended ones disagree (different-trial). This means that attended and non-attended halves are *congruent* with respect to the correct decision. In incongruent trials, however, the unattended halves disagree when the attended halves agree (same-trial), and agree when the attended ones disagree (different-trial). Hence, attended and unattended halves are *incongruent* with regard to the correct decision. Holistic effects are operationally defined as *congruency effects*, reflecting the performance difference achieved in congruent and incongruent trials (see Performance Measures)^1^.

### 2.3. Stimuli

**Figure 3:**
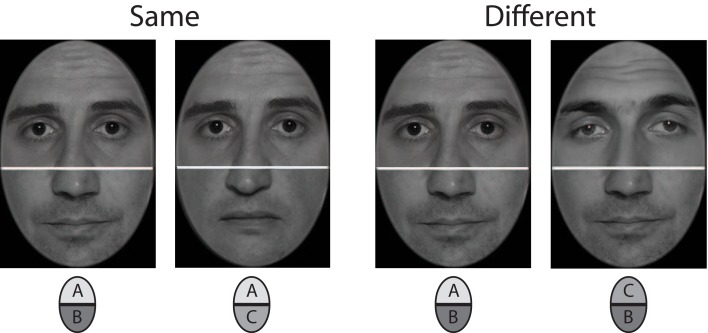
**Stimulus example for upper face half comparison in incongruent trials (mid row of Figure 2)**. The left composite face pair shows same upper halves combined with different lower halves, the right one shows different upper halves combined with same lower halves.

Photographs of 20 male models were used for stimulus construction (see Figure 3 for examples). These were frontal view shots of the whole face, captured in a professional photo studio under controlled lighting conditions. The original images were edited with Adobe Photoshop CS4 to generate the set of stimuli used in the experiment. Photographs were initially converted to 8 bit grayscale pictures and superimposed with an elliptical frame mask to obliterate all external facial features such as hair, ears, or chin line. The elliptical cutouts were then split horizontally at the bridge of the nose, thus yielding 20 upper and 20 lower face halves. Each upper half was recombined with three lower halves to constitute the final set of 60 compound faces. The cutline between the face halves was concealed with a white bar of 5 pixels thickness. It was warranted that any upper face part was never recombined with the lower half of the same original face. In addition, each of the twenty lower and upper halves appeared exactly three times in the final set of stimuli. Stimulus size was 250 × 350 pixels (width × height), which corresponded to 10 × 12.5 cm of the screen. For each face stimulus a corresponding mask was constructed by sampling randomly ordered 5 × 5 pixel blocks from the face image. Masks subtended 350 × 450 pixels (width × height), and covered the whole region where two subsequent face stimuli were displayed.

### 2.4. Subjects

Overall, 46 young adults and 40 senior subjects participated in the present study. The two samples were halved, one group participated in the experiment with feedback, the other without feedback. All participants had normal or corrected to normal vision and reported normal neurological and psychiatric status. Senior subjects lived independent lives and were paid for participation.

The mini-mental state examination (MMSE; Folstein et al., 1975) was used to evaluate mental status. Young adult subjects were undergraduate students, 20% were male and 80% female. The mean age of the student group was 23.1 (range 20–32). These participants were given course credit points for participation, or received payment. Senior subjects were assigned to the feedback and no-feedback groups in a pseudo-random procedure with the constraint to keep the age structure of the groups equivalent. Feedback group: 20 subjects (11 female; mean age = 69.7; range 65–78 years), and No feedback group: 20 subjects (14 female; mean age = 70.1; range 65–77 years). All subjects were naive with respect to the purpose of the experiment. The study was conducted in accordance with the Declaration of Helsinki. In detail, subjects participated voluntarily and gave written informed consent to their participation. In addition, participants were informed that they were free to stop the experiment at any time without negative consequences. The data were analyzed anonymously.

### 2.5. Apparatus

The experiment was executed with Inquisit runtime units. Stimuli were displayed on NEC Spectra View 2040 TFT displays in 1280 × 1024 resolution at a refresh rate of 60 Hz. Screen mean luminance *L*_0_ was 100 cd/m^2^ at a michelson contrast of (*L*_*max*_ − *L*_*min*_)/(*L*_*max*_ + *L*_*min*_) = 0.98, therefore the background was practically dark (about 1.4 cd/m^2^, measured with a Cambridge Research Systems ColorCAL colorimeter). No gamma correction was used. The room was darkened so that the ambient illumination approximately matched the illumination on the screen. Stimuli were viewed binocularly at a distance of 70 cm. Subjects used a distance marker but no chin rest throughout the experiment. Stimuli were viewed at 70 cm viewing distance. Subjects responded via an external key-pad, and wore light headphones for acoustical feedback in the feedback condition.

### 2.6. Preparation and preliminary measurements

Preliminary measurements were taken with four senior subjects to assure that the task could, in principle, be executed by the elderly, and to determine the proper exposure durations for the test stimuli. Several exposure durations were probed to find a relaxed timing that allowed for maximum performance of senior subjects under the experimental conditions with the lowest attentional and perceptual demands (i.e., for the target cue with the study image, providing feedback, and for congruent trials). It turned out that senior subjects could respond to these trials with about 90% correctness at test stimulus exposure durations of half a second and longer.

Enlarging exposure duration to about a second did not increase accuracy any further. Note that 90% correct corresponded to only three errors out of 32 replications. We then presented incongruent and congruent trials mixed in random order, which did not lead to a stronger decline in accuracy for the congruent trials when exposure durations of well beyond 500 ms were used. We decided to use 633 ms (36 frames of the monitor at 60 Hz refresh rate) as the largest exposure duration.

### 2.7. Procedure

Subjects were informed that face pairs could differ in the cued halves, but also in non-cued halves, and face halve comparison was to be done for just the cued halves. They were also instructed to compare the face halves as accurately as possible, without speed pressure for the response. The temporal order of events in a trial sequence was: fixation mark (750 ms), blank (300 ms), study face stimulus (800 ms), mask (400 ms), blank (800 ms), test face stimulus (50, 233, or 633 ms), mask (400 ms), and blank frame until response (see Figure 1).

In the *1st cue* condition a rectangular bracket marking the target face half was shown simultaneously with the study face, and remained until the test face was masked. In the *2nd cue* condition the cue presentation began with the mask of the study face. Stimulus position jittered randomly within a region of ±50 pixels around the center of the screen to preclude image region matching strategies between two subsequent stimulus presentations.

Young adults were made familiar with the task by going through randomly selected probe trials to ensure that the instruction was understood and could be put into practice. Senior subjects were carefully prepared for the experiment. First, the researcher explained the sequential composite face task using paper print examples of the stimulus pairings. To ensure that subjects understood the composite face task with incongruent face halve pairings, the experimenter displayed paper prints of 10 stimulus pairs, and asked participants to name the five pairs showing objects with the same upper (lower) halves and five showing different upper (lower) halves. Subjects were given as much time as needed to label the 10 pairs. If errors occurred, the experimenter adverted to the wrongly labeled pairs and drew attention to just the halves to be compared. The first minutes at the computer were spent on just congruent trials presented with the longest viewing time (633 ms), which all subjects could do with good accuracy. They then saw probe trials of the experiment with congruent and incongruent trials for about 8 min. After the preparation phase, the experimental blocks started.

Each subject went through 2 (cue position) × 2 (congruency) × 3 (duration) = 12 conditions. Each condition was measured with 16 same- and 16 different- trials. Eight of these 16 replications were done with upper half, and 8 with lower half as the target, resulting in 384 trials. These were subdivided into a block of 192 trials where the target cue came at the first position and a block of 192 trials where the cue came at the second position. Going through a block took about 20 min. Interleaved by a brief pause, the two blocks were administered on a single day, one with 1st cue, and one with 2nd cue, in random order across subjects.

### 2.8. Performance measures

Accuracy was measured in terms of the proportion of correct judgments, *P*_*c*_. The rates were calculated from the frequencies of correct “same” [*h*_*S*_] and correct “different” [*h*_*D*_] judgments, i.e., *P*_*c*_ = (*h*_*S*_ + *h*_*D*_)/(*n*_*S*_ + *n*_*D*_). With *n*_*S*_ = *n*_*D*_ = 16 replications per trial, each proportion correct datum rested on *n* = 32 trials. Congruency effects were calculated as the difference

(1) $$CE=P_{c}(congruent)-P_{c}(incongruent).$$

Originally, Cheung et al. (2008) referred to the *d*′ measure as a bias-free measure. We used the proportion correct measure, because, as *d*′, proportion correct also derives from the performance achieved for both response alternatives. However, it avoids hypothetical assumptions about sensory mapping of face stimuli, and the distribution of the corresponding sensory states. Further, it reflects task difficulty on a direct and intuitive scale.

Moreover, a direct and intuitive measure of response bias can be defined by referring to the relative frequencies for the errors of both kinds (Meinhardt-Injac et al., 2014a). For the same/different experiment the “same” response category is commonly defined as the target category (e.g., Richler et al., 2011). Accordingly, hit-rate (Hit) was defined as the rate of correctly identifying same target halves and correct rejection rate (CR) was defined as the rate of correctly identifying different target halves. False alarm rate (FA) and the rate of misses (Miss) were defined as being the complementary rates to CR and Hit, respectively. We measured response bias in terms of the error proportion, *Q*, which indicates which of both errors is more likely:

(2) $$Q=\frac{Miss}{Miss+FA}.$$

If *Q* = 0.5, then both kinds of errors are made with the same frequency. A ratio of *Q* > 0.5 indicates a tendency to say “different” while *Q* < 0.5 indicates a preference toward “same” responses. The *Q*- measure has the advantage that it easy to interpret. For example, a value of *Q* = 0.7 means that 70% of all errors are wrong “different” responses and 30% are wrong “same” responses^2^.

### 2.9. Data analysis

The proportion correct data and the *Q*- measure were analyzed with ANOVA, having feedback (FB) and age group (Age) as grouping factors and cue position (Cuepos), congruency (Congru) and exposure duration (Time) as repeated measurement factors. We do not report ANOVA results for the CE measure, since the results for the difference measure are already included in the results for all interactions involving congruency at the original *P*_*c*_ data.

## 3. Results

**Figure 4:**
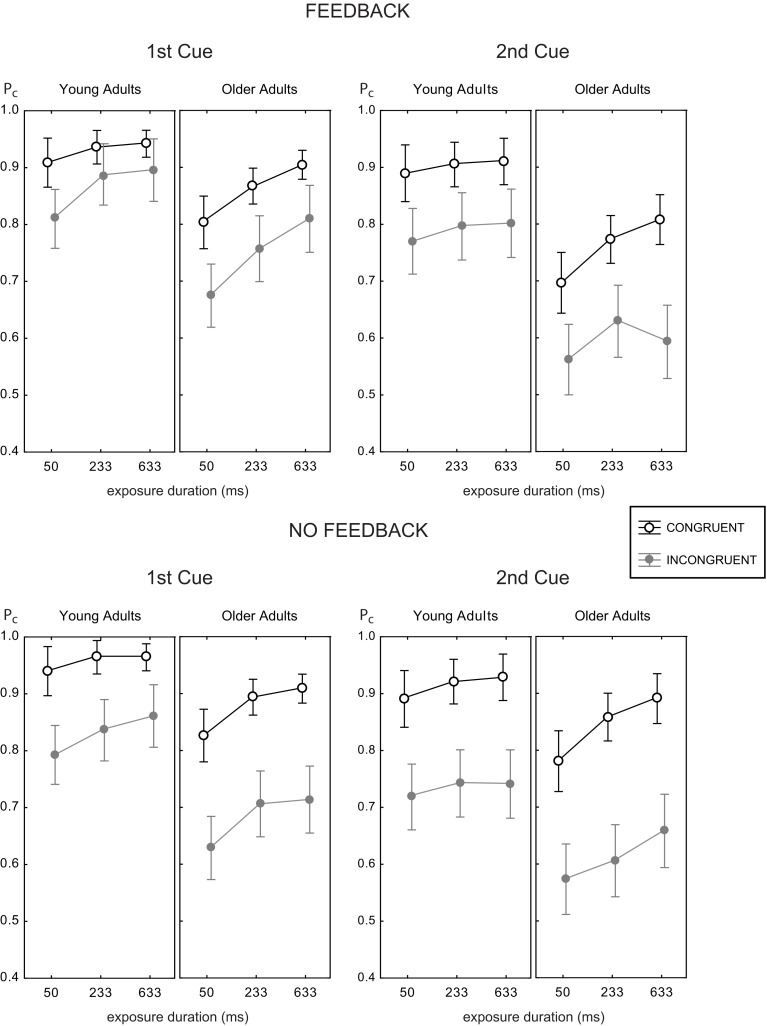
**Mean proportion correct rates as a function of exposure duration for the two age groups with feedback (upper panels) and without (lower panels), and target face half cue given at study image (1st cue, left panels) and before test image (2nd cue, right panels)**. Data for the congruent trials are shown as open black circles, gray symbols indicate data for incongruent trials. Error bars indicate 95% confidence limits of the means.

Figure 4 shows the mean proportion of correct responses as a function of exposure duration for all experimental conditions. Generally, both younger and older adults reached good accuracy levels above 90% correct at intermediate (233 ms) and large (633 ms) viewing times for congruent trials. For incongruent trials, performance did not come close to these levels, and even declined. Hence, a large congruency effect was found in all experimental conditions, which became obvious by the space between the black and gray curves.

Data analysis using ANOVA revealed main effects of exposure duration [*F*_(3, 164)_ = 69.08, *p* < 0.001], congruency [*F*_(1, 82)_ = 191.10, *p* < 0.001], cue position [*F*_(1, 82)_ = 89.65, *p* < 0.001], and age group [*F*_(1, 82)_ = 64.04, *p* < 0.001], but no main effect of feedback [*F*_(1, 82)_ = 3.02 × 10^−4^, *p* = 0.986]. We explored these effects further explored by analyzing first and higher order interactions.

### 3.1. Effects of feedback and cue position

**Figure 5:**
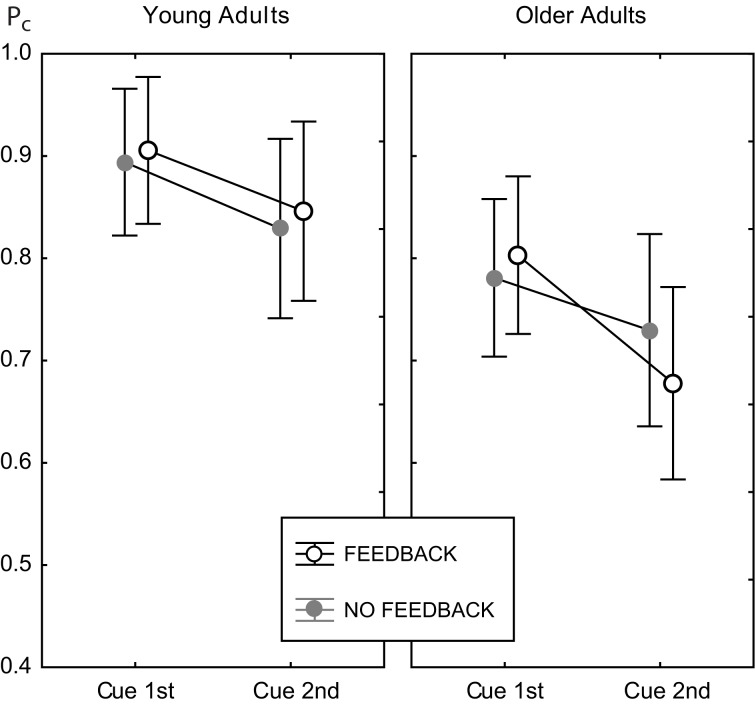
**Cell means plot for the proportion correct measure, illustrating the cue position × feedback × age group interaction**. Data for the feedback condition are shown as open black circles, gray filled circles indicate data for the no feedback condition. Error bars indicate 95% confidence limits of the means.

There was no main effect of feedback, and no interaction of feedback with age [*F*_(1, 82)_ = 0.62, *p* = 0.434]. Hence, feedback did not change the general level of performance in both age groups. However, feedback substantially modified the effect of congruency [congruency × feedback, *F*_(1, 82)_ = 10.18, *p* < 0.002, see below], and the effect of cue position [cue position × feedback, *F*_(1, 82)_ = 4.66, *p* < 0.04]. However, the latter was further moderated by age group [cue position × feedback × age group, *F*_(1, 82)_ = 6.28, *p* < 0.02]. Figure 5 illustrates this interaction.

For young adults the effects of feedback were the same in both cue positions. For older adults, performance in the 2nd cue condition was disproportionately worse *with* feedback. This finding was confirmed by pairwise Fisher LSD *post-hoc* tests. Older adults did not perform significantly different in both feedback conditions at the 1st cue position (Δ*P*_*c*_ = 0.023, *p* = 0.373), but significantly worse with feedback at the 2nd cue position (Δ*P*_*c*_ = 0.052, *p* < 0.04). Exploring the role of trial type showed the same performance for both feedback conditions in incongruent trials (Δ*P*_*c*_ = 0.019, *p* = 0.582), but worse performance with feedback in congruent trials (Δ*P*_*c*_ = 0.085, *p* < 0.02; see also right panels of Figure 4). At the 1st cue position older adults performed better with feedback than without in incongruent trials (Δ*P*_*c*_ = 0.064, *p* < 0.05), and the same in both feedback conditions in congruent trials (Δ*P*_*c*_ = 0.018, *p* = 0.328). This finding indicates a paradox effect of feedback in the old age group for the condition with high attentional demand. For young adults, the same results scheme for the effects of feedback was found for the 1st cue and the 2nd cue position. These participants performed better with feedback in incongruent trials (1st cue: Δ*P*_*c*_ = 0.041, *p* < 0.05; 2nd cue: Δ*P*_*c*_ = 0.054, *p* < 0.04) and the same with and without feedback in congruent trials (1st cue: Δ*P*_*c*_ = 0.015, *p* < 0.40; 2nd cue: Δ*P*_*c*_ = 0.02, *p* = 0.516).

Figure 5 illustrates that cue position modified performance strongly, which led to significantly lower performance in the 2nd cue condition. The effect of cue position was not modulated by age [cue position × age group, *F*_(1, 82)_ = 2.71, *p* = 0.104], however, it was by age and feedback (see above). Table 1 shows the effects of cue position for both age groups and feedback conditions and their effect sizes (Cohen's *d* effect size measure). The data show that cue position had large effects of comparable sizes in both age groups in the no feedback condition. Adding feedback did not affect much for young adults, but more than doubled the effect for older adults, both in the accuracy measure, and in effect size.

**Table 1:** **Effects of cue position for the two age groups and feedback conditions**.

**Age group**	**Feedback**	**Δ*****P***_***c***_	***F***_**(1, 82)**_	***p***	***d***
Older adults	No FB	0.051	9.72	0.003	0.70
Older adults	FB	0.125	58.18	0.001	1.71
Young adults	No FB	0.065	17.94	0.001	0.88
Young adults	FB	0.059	15.02	0.001	0.81

The table shows the accuracy difference for 1st and 2nd cue position, *F* - value, significance level, and Cohen's *d* measure.

### 3.2. Congruency effects

Variation of the congruency relation (congruent/incongruent) among face halves strongly modulated performance. With respect to age group we found larger congruency effects for older adults [congruency × age group, *F*_(1, 82)_ = 5.34, *p* < 0.02]. Comparing across age for congruent and incongruent trials separately with Fisher LSD *post-hoc* tests showed that young adults were better than older adults particularly in incongruent trials (congruent: Δ*P*_*c*_ = 0.096, *p* < 0.001; incongruent: Δ*P*_*c*_ = 0.146, *p* < 0.001). This finding indicates age-related differences in the ability to suppress incongruent facial context.

Feedback strongly modified the effect of congruency [congruency × feedback, *F*_(1, 82)_ = 10.18, *p* < 0.002]; the congruency effect was strongly attenuated when feedback was provided, which is readily seen when the space between the black and the gray curves shown in Figure 4 is compared among the upper and the lower data panels. Further, cue position strongly modulated the congruency effect [cue position × congruency, *F*_(1, 82)_ = 13.48, *p* < 0.001], which reflects larger congruency effects for the 2nd than for the 1st cue position.

Interestingly, no higher interactions were found with age group, indicating that the congruency effect was modulated by feedback and cue position in the same way for younger and older adults [congruency × feedback × age group, *F*_(1, 82)_ = 0.05, *p* = 0.819; cue position × congruency × age group, *F*_(1, 82)_ = 0.03, *p* = 0.853]. Table 2 lists the congruency effects of both age groups, for both cue positions and feedback conditions. The data reflect that older adults had consistently larger congruency effects than did the young adults, in the order of magnitude of 5% (see last column). The table also shows that the modulating effects of feedback and cue position on the congruency effect were the same in both age groups, and in the range of 4–6% (cue position), and 5–8% (feedback), respectively.

**Table 2:** **Congruency effects for the two target cue positions and the two feedback conditions, for both age groups**.

**Age group**	**Feed back**	**Cuepos**	***CE***	**Δ (Cuepos)**	**Δ (Feedback)**	**Δ (Age)**
Older adults	No FB	Cue 2nd	0.231	0.038	0.066	0.043
Older adults	No FB	Cue 1st	0.193		0.082	0.067
Older adults	FB	Cue 2nd	0.165	0.053		0.052
Older adults	FB	Cue 1st	0.111			0.038
Young adults	No FB	Cue 2nd	0.188	0.061	0.075	
Young adults	No FB	Cue 1st	0.126		0.053	
Young adults	FB	Cue 2nd	0.112	0.039		
Young adults	FB	Cue 1st	0.073			

The table shows mean congruency effects, the difference of the mean congruency effects for both cue positions, both feedback conditions, and the difference of congruency effects across age group for the same conditions.

### 3.3. Effects of exposure duration

The effect of exposure duration was different in the two age groups [exposure duration × age group, *F*_(2, 164)_ = 9.14, *p* < 0.001], with smoothly rising performance across viewing times for young adults, while performance showed stronger improvement with viewing time for older adults. There were no time-related effects of feedback, cue position, or congruency, which indicates that all these effects were relatively constant across exposure duration. There was time related effect that concerned the congruency effect at the two cue positions [cue position × congruency × exposure duration, *F*_(2, 164)_ = 3.45, *p* < 0.04]. This effect reflected that congruency effects tended to decline with increasing viewing time when the cue came at the 1st position, while congruency effects tended to increase with exposure duration when the cue came at the 2nd position. No age-related differences were indicated for this effect by statistical testing [cue position × congruency × exposure duration × age group, *F*_(2, 164)_ = 0.29, *p* = 0.746].

### 3.4. Response bias

**Figure 6:**
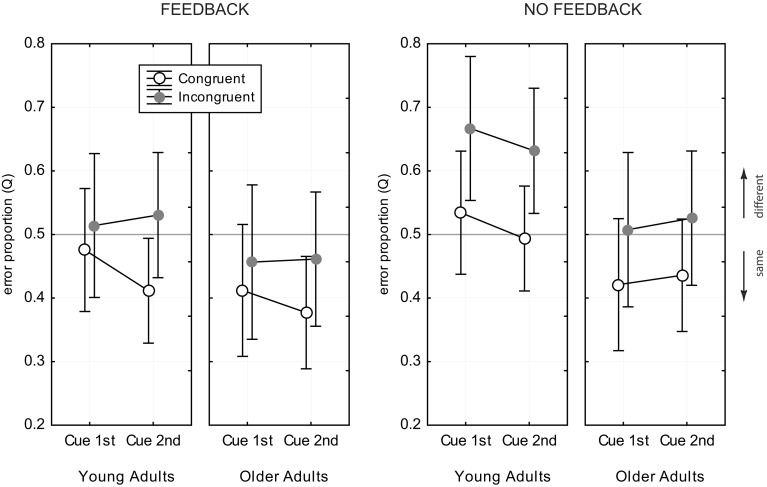
**Error proportion, *Q*, for assessing response bias**. Data for congruent trials are shown as open black circles, gray filled circles indicate data for incongruent trials. Values of *Q* > 0.5 indicate a bias toward “different” responses, and values of *Q* < 0.5 toward “same” responses. Error bars indicate 95% confidence limits of the means.

Figure 6 shows the data for the *Q*- measure. ANOVA revealed main effects of age group [Δ*Q* = 0.08, *F*_(1, 82)_ = 12.05, *p* < 0.001], congruency [Δ*Q* = 0.09, *F*_(1, 82)_ = 101.6, *p* < 0.001], and feedback [Δ*Q* = 0.07, *F*_(1, 82)_ = 9.55, *p* < 0.001], but no effects of cue position [*F*_(1, 82)_ = 1.38, *p* = 0.24] and exposure duration [*F*_(1, 82)_ = 0.36, *p* = 0.70]. Young adults tended to prefer the “different” response category [*Q* = 0.53, *CI* = [0.50, 0.56]], while older adults preferred “same” responses [*Q* = 0.45, *CI* = [0.42, 0.48]]. The *Q*- measure was consistently larger in incongruent trias, compared to congruent trials [*Q*(*IC*) = 0.54, *CI* = [0.51, 0.56], *Q*(*CC*) = 0.45, *CI* = [0.42, 0.47]], and also consistently larger in the no feedback condition, compared to the feedback condition [*Q*(*NoFB*) = 0.53, *CI* = [0.49, 0.56], *Q*(*FB*) = 0.45, *CI* = [0.42, 0.48]]. There was a significant interaction of congruency and feedback [*F*_(1, 82)_ = 4.87, *p* < 0.03], which indicated that the difference in the *Q*- measure for congruent and incongruent trials was stronger in the no feedback condition, compared to the feedback condition (see Figure 6). Young adults showed a strong response bias toward “different” responses in incongruent trials when there was no feedback (see Figure 6). The bias vanished when feedback was provided. Older adults did not prefer “different” responses in any experimental condition.

## 4. Discussion

We studied holistic face perception with the complete design of the composite face paradigm, to explore the particular role of attentional demand, feedback, and viewing time, and to compare these factors across younger and older adults. Younger adults could do the study-test composite face task at brief timings (50 ms exposure duration) and at good performance levels. Older adults started at lower levels for the shortest timing, but well above chance, and reached good performance of about 90% accuracy at relaxed viewing times (633 ms).

We obtained strong congruency effects in all experimental conditions, which were consistently larger for older adults. Age-related differences were particularly pronounced for incongruent trials. However, the modulation of congruency effects by feedback and attentional demand was highly similar in both age groups. Generally, congruency effects were strongest when subjects were forced to change their attentional focus within a trial, and when no feedback was provided. A strong interaction of attentional demand, feedback, and age group was observed. Young adults could exploit trial-by-trial feedback to improve performance in incongruent trials with high and low attentional demand. Older adults could do so only in trials with low attentional demand. When participants were forced to reallocate attentional resources within a trial, performance was worse with feedback than without.

Analysis of response bias revealed a tendency of older adults toward preferring “same” responses, while young adults were slightly biased toward “different” responses. Feedback led toward more frequent “same” responses in both age groups.

### 4.1. No age-related decline in congruency effects

One aim of this study was to re-examine age-related changes in the congruency effect as an important hallmark of perceptual integration in face perception. We obtained consistently larger congruency effects for the elderly in all experimental conditions. Further, the strong performance difference of congruent and incongruent trials was observed in both age groups at brief timings of 50 ms, and remained for more relaxed timings. Hence, no indication was found of age-related decline in the general capabilities to view faces holistically. In line with recent results (Daniel and Bentin, 2012; Konar et al., 2013; Meinhardt-Injac et al., 2014b), our results suggest the elderly prefer global and holistic viewing strategies, albeit part-based viewing strategies are more effective for task success.

### 4.2. Effects related to task demands

Face half comparisons are more difficult in the 2nd cue condition, since a late cue enforces fast reallocation of resources (Greenwood and Parasuraman, 1999; Georgiou-Karistianis et al., 2006). A second reason for higher task difficulty in the late cue condition is enhanced demand for encoding and fast retrieval from working memory. When the cue comes with the study image observers can encode only the face half of interest and compare it to the target test half, while trying to ignore the non-target half. When the cue comes late it is not possible to proceed this way, and the observers must encode information of both halves at study.

Both attentional control and working memory are known to be affected by aging. Several studies have shown that the elderly operate much worse than young adults in tasks that require attentional switch (Lincourt et al., 1997; Greenwood and Parasuraman, 1999; Vanneste and Pouthas, 1999; Georgiou-Karistianis et al., 2006). Using Navon-like stimuli and task (Navon, 1977), Georgiou-Karistianis and colleagues showed that older adults exhibited a similar global precedence effect as young adults, but they performed worse when a switch from global to local or from local to global was required. In contrast, young adults exhibited only moderate or no switching costs. Age-related decline in working memory is a well-established finding that is substantiated by many studies (for a review, see Rajah and D'Esposito, 2005). Both the decline in working memory function and loss of attentional control can be understood within the framework of the frontal lobe hypothesis of aging (West, 1996), because divided attention, attentional and executive control, and working and episodic memory were found to be mediated by frontal brain areas (Goldman-Rakic, 1995; Cabeza et al., 1997; Fink et al., 1997; Rajah and D'Esposito, 2005; Prakash et al., 2009). From these results it can be expected that the combined effects of higher attentional demands and stronger working memory requirements in the late cue condition should disproportionately affect the performance of older adults. Interestingly, our results do *not* support a disproportionate age-related decline of performance in the 2nd cue condition.

As outlined in the Results section (see Figure 5 and Table 1) the effect of cue position was the same in both age groups, as long as there was no feedback. The effect of cue position was larger for older adults *only* in the feedback condition, for specific reasons (see below). Hence, the increase of task demands in the 2nd cue condition compared to the 1st cue condition affected performance of young and older adults to the same degrees. This finding indicates that younger and older adults handled increased task demands equally well. In view of the fact that cue position modulated task difficulty strongly, this finding is at odds with expectation from the known aging effects on working memory function and attentional control.

We also found that the effect of cue position on the congruency effect was not different for young and older adults (see Congruency Effects). Increased task demands strengthened the influence of the unattended face halves, in the same way for both age groups. The surprising fact that both performance and congruency effects of older adults were not disproportionately affected by the much higher task demands in the late cue condition points to a potential benefit of holistic encoding, which might have been used as a strategy. Holistic encoding spares the costs of divided attention to lower and upper halves at study, which precludes the effects of restricted capabilities in divided attention to become effective (Greenwood and Parasuraman, 1999). However, the encoding advantage is at the costs of having to recall the diagnostic features of just one half from a holistic representation, which results in stronger interference among target the half and incongruent non-target half. Accordingly, an increase of contextual interference for 2nd cue trials should result, which was indeed observed.

The composite face task was generally much more difficult for the elderly, as indicated by the strong main effect of age. One likely reason why face comparisons were more difficult for older adults is the use of elliptical frames that leave only the inner face parts and mask global face shape and further external features. Meinhardt-Injac et al. (2014b) used full and intact face stimuli, and had subjects attend to either the internal or external features. They found that older adults were nearly as good as young adults in comparing external features, but were much worse when internal features were the focus. This finding indicates that global face shape is a relevant face identity cue for the elderly (see below).

### 4.3. Response bias

A considerable advantage of the CD compared to the PD is that the CD is fully balanced with respect to congruency relation and the number of same and different face halves (Richler et al., 2011). Thus, the CD avoids that response bias is induced due to methodological artifacts. Analysis of response preferences can therefore reveal true age-related differences, as well as influence of experimental conditions on decision behavior. In this study we found evidence for different response behavior in both age groups, and modulatory influence of feedback and congruency relation, but no influence of task demands and exposure duration. Young adults strongly preferred the “different” response in incongruent trials when there was no feedback. The bias toward “different” responses was found in several studies using the CD (Cheung et al., 2008; Richler et al., 2008a; Gao et al., 2011), and might indicate that the difference of the wholes and the unattended parts bias the observer toward responding “different,” albeit the attended parts are same (Gao et al., 2011). Interestingly, trial-by-trial feedback canceled this effect. With the help of feedback young observers noticed that they relied on the wrong features, and they could revise their decisional strategy. This is in line with the observation that feedback helped to improve young adults' performance in incongruent trials. Older adults, in contrast, did not show a “different” bias in any experimental condition. While they responded “different” more often in incongruent trials, compared to congruent trials, they stayed generally biased toward “same” responses. With feedback the overall preference toward “same” responses even increased. The general “same” bias might indicate that elderly tend to overlook local diagnostic features that are crucial for facial comparisons. This is supported by earlier and recent findings which show that older adults tend to more likely identify new faces as previously seen ones (Bartlett et al., 1989; Fulton and Bartlett, 1991; Lee et al., 2014). In a recent aging study of Konar et al. (2013) no response bias was found for young and older adults. However, the authors used the PD and concluded holistic processing from the difference achieved with aligned and misaligned presentation. This might account for differences of their results and the findings of this study.

### 4.4. The paradox effect of feedback in the older adults group

Perceptual learning studies have found that feedback enables observers to revise and to optimize their viewing strategies (Herzog and Fahle, 1997, 1999). Face perception studies have also found that young adults identify diagnostic facial features and regulate the influence of irrelevant context with the help of feedback (Meinhardt-Injac et al., 2011). The results obtained here show that feedback had exactly this effect for young and older adults, as long as task demands were moderate. In the late cue condition young adults were still able to benefit from feedback, particularly in the incongruent trials. In contrast, the performance of older adults was not better with feedback in incongruent trials, while performance in the easier congruent trials declined (see Results). Seemingly, older adults were confused by the feedback signal in the late cue condition, and failed to establish a correlation of strategy revision and success. At the same time, the lower performance levels of older adults indicate that they experienced high task difficulty (see Figure 5). This finding corresponds to an interaction of task difficulty and learning observed in perceptual learning (Ahissar and Hochstein, 1997, 2004). When task difficulty is high, learning usually does not occur, even when external markers are provided. Subjects need some easy trial instances to initiate learning (“eureka effect,” see Ahissar and Hochstein, 2004). Hence, the inability to benefit from feedback in the condition with the highest task demands may indicate an interaction of learning and task difficulty for the elderly. This effect should not be over-estimated, as it is observed for the first time in the context of the composite face task. However, it would be interesting to see whether the effect is also obtained with non-face objects because older adults do not seem to apply global viewing strategies (Meinhardt-Injac et al., 2014b). As the stronger congruency effects for older adults indicate, it is adherence to global viewing strategies that is in conflict with feedback. The difficulties of elderly to replace a global viewing strategy with a more effective piecemeal strategy when task demands are high is in line with recent claims that older adults use holistic processing as a strategy to reduce perceptual and cognitive load (Dror et al., 2005; Konar et al., 2013).

### 4.5. How do elderly look at faces?

Looking at the composite effects for the elderly (see Table 2) shows that the influence of unattended face halves in the feedback condition is still as great as for young adults in the no feedback condition. Therefore, the general level of contextual influence remains high for older adults, even in conditions that are optimal for setting up a piecemeal viewing strategy.

The large global-contextual influence for older adults indicates that age-related decline in face perception does not concern mechanisms of perceptual integration. Rather, the elderly suffer from deficits when analytical processing of faces and control of facial context is required. Further evidence that face-specific processing is intact in older adults comes from the face inversion effect (FIE, Yin, 1969). Comparing across the life span, Germine et al. (2011) reported that the FIE in a face recognition task gradually increases up to ages 62 years, indicating that the experience dependent advantage of upright face processing is not lost in mature ages. Murray et al. (2010) found that elderly were much more vulnerable to face rotation than were young adults, which indicates that they strongly rely on configural information of facial features. Similar findings were reported by Creighton et al. (submitted). For older adults accuracy, response latency, and intensity rating for facial expressions of anger, happiness, fear and sadness were notably impaired when faces were turned upside down. Inversion effects for young adults were much smaller (fear, sadness) or even absent (anger, happiness).

Comparing the FIE for horizontal (eye distance) and vertical (eye-mouth distance) relational face manipulations across age, Chaby et al. (2011) observed that the FIE for vertical-relational manipulations was preserved in the elderly, while the FIE for horizontal-relational manipulations was lost. However, the overall accuracy level was lower than for young adults in detecting vertical relational changes. Obermeyer and colleagues obtained similar findings concerning age-related decline in face recognition with images that contained only horizontal spatial frequency information (Obermeyer et al., 2012). They also found a strong FIE of more than one *d*′ unit in both age groups for this type of image manipulation. The strong FIE for vertical-relational manipulations, together with the loss of the FIE for horizontal-relational manipulations is diagnostic of the facial cues preferred by older adults. Eye distance (horizontal) is a local-relational feature judged relatively independent of facial context (Leder et al., 2001). In contrast, eye height (vertical) is defined in terms of its distance to the mouth, forehead and face outline, and is a global, long-range relational feature (Sekunova and Barton, 2008; Meinhardt-Injac et al., 2011). Chaby et al. (2011) reported a strong age-related decline in assessing local-configural facial features, while global-configural features could still be assessed. This finding is in-line with Daniel and Bentin (2012), who recorded the face specific N170 potential and the P300 component to reveal global, configural and featural face-processing strategies. Daniel and Bentin (2012) found that older adults relied on distal global information, and tended to process faces merely at the basic level of categorization until identification was required. Moreover, the elderly did not apply configural information by default, and showed deficits in subordinate categorization (gender classification based on internal features), which strongly relies on local-configural cues. Recent results from sequential same/different tasks with whole or just part-based agreement in external and internal features showed that the elderly rely more on global shape information than do young adults, and they experience deficits in judging inner face details (Meinhardt-Injac et al., 2014b). Also the finding of a global bias toward “same” responses indicates that elderly have difficulties to focus the diagnostic features when they compare faces. These results, together with the findings of a less flexible handling of viewing strategies show that the elderly generally process faces holistically, but suffer from losses in assessing local-configural features, particularly when maintenance of attentional focus is impeded by the complexity of the visual task.

## Author contributions

All authors contributed equally to the conceptualization of the study. Bozana Meinhardt-Injac set up the basic design. Malte Persike conducted the experiments and data preparation. Günter Meinhardt contributed data analysis and interpretation. All authors were involved in writing, preparation of the manuscript and final approval. All authors agree to be accountable for all aspects of the work in ensuring that questions related to the accuracy or integrity of any part of the work are investigated and resolved appropriately.

### Conflict of interest statement

The authors declare that the research was conducted in the absence of any commercial or financial relationships that could be construed as a potential conflict of interest.

## Footnotes

^1^We chose the complete design because of its methodological advantages (see Cheung et al., 2008; Richler et al., 2011). In the PD same-trials are used only with incongruent pairings of upper and lower halves, while different-trials are used only with congruent relations. This means that different-trials are, in principle, easier than same-trials, and might lead to artificial composite effects because they are concluded from the relative frequency observed for erroneously classifying same-trials as different. Further, different face halves occur more frequent than same face halves, which might induce a response bias toward “different” responses. In criticism of the PD, Cheung and colleagues proposed to measure the composite effect in terms of the congruency effect, which considers the judgments for both congruency relations and both response categories. For a discussion of the design issue see Richler and Gauthier (2013) and Rossion (2013), for an alternative position.

^2^Results in terms of *d*′ and the response criterion, *c*, both calculated using standard formulae, can be found in the Supplementary Material.

## References

[B1] Ahissar M. Hochstein S. H. (1997). Task difficulty and the specificity of perceptual learning. Nature 378, 401–406. 10.1038/387401a0 9163425

[B2] Ahissar M. Hochstein S. H. (2004). The reverse hierarchy theory of visual perceptual learning. Trends Cogn. Sci. 8, 457–464. 10.1016/j.tics.2004.08.011 15450510

[B3] Bartlett J. C. Leslie J. E. Tubbs A. Fulton A. (1989). Aging and memory for pictures of faces. Psychol. Aging 4, 276–283. 10.1037/0882-7974.4.3.276 2803620

[B4] Boutet I. Faubert I. (2006). Recognition of faces and complex objects in younger and older adults. Mem. Cogn. 34, 854–864. 10.3758/BF03193432 17063916

[B5] Brockmole J. R. Logie R. H. (2013). Age-related change in visual working memory: a study of 55,753 participants aged 8–75. Front. Psychol. 4:12. 10.3389/fpsyg.2013.00012 23372556 PMC3557412

[B6] Cabeza R. Grady C. L. Nyberg L. McIntosh A. R. Tulving E. Kapur S.. (1997). Age-related differences in neural activity during memory encoding and retrieval: a positron emission tomography study. J. Neurosci. 17, 391–400. 8987764 10.1523/JNEUROSCI.17-01-00391.1997 PMC6793692

[B7] Chaby L. George N. Renault B. Fiori N. (2003). Age-related changes in brain responses to personally known faces: an event-related potential (erp) study in humans. Neurosci. Lett. 349, 125–129. 10.1016/S0304-3940(03)00800-0 12946568

[B8] Chaby L. Narme P. George N. (2011). Older adults' configural processing of faces: role of second-order information. Psychol. Aging 26, 71–79. 10.1037/a0020873 20973603

[B9] Cheung O. S. Richler J. J. Palmeri T. J. Gauthier I. (2008). Revisiting the role of spatial frequencies in the holistic processing of faces. J. Exp. Psychol. Hum. Percept. Perform. 34, 1327–1336. 10.1037/a0011752 19045978

[B10] Crook T. H. Larrabee G. J. (1992). Changes in face recognition memory across the adult life span. J. Gerontol. 47, 138–141. 10.1093/geronj/47.3.P138 1573194

[B11] Daniel S. Bentin S. (2012). Age-related changes in processing faces from detection to identification: Erp evidence. Neurobiol. Aging 33, 206.e1–28. 10.1016/j.neurobiolaging.2010.09.001 20961658 PMC3025306

[B12] Dror I. E. Schmitz-Williams I. C. Smith W. (2005). Older adults use mental representations that reduce cognitive load: mental rotation utilizes holistic representations and processing. Exp. Aging Res. 31, 409–420. 10.1080/03610730500206725 16147460

[B13] Fink G. Halligan P. Marshall J. Frith C. Frackowiak R. Dolan R. (1997). Neural mechanisms involved in the processing of global and local aspects of hierarchically organized visual stimuli. Brain 120, 1779–1791. 10.1093/brain/120.10.1779 9365370

[B14] Folstein M. F. Folstein S. E. McHugh P. R. (1975). “Mini-mental state.” A practical method for grading the cognitive state of patients for the clinician. J. Psychiatr. Res. 12, 189–198. 1202204 10.1016/0022-3956(75)90026-6

[B15] Fulton A. Bartlett J. C. (1991). Young and old faces in young and old heads: the factor of age in face recognition. Psychol. Aging 6, 623–630. 10.1037/0882-7974.6.4.623 1777151

[B16] Gao Z. Flevaris A. V. Robertson L. C. Bentin S. (2011). Priming global and local processing of composite faces: revisiting the processing-bias effect on face perception. Attent. Percept. Psychophys. 73, 1477–1486. 10.3758/s13414-011-0109-7 21359683 PMC3118009

[B17] Gauthier I. Bukach C. (2007). Should we reject the expertise hypothesis? Cognition 103, 322–330. 10.1016/j.cognition.2006.05.003 16780825

[B18] Gazzaley A. Clapp W. Kelley J. McEvoy K. Knight R. T. D'Esposito M. (2008). Age-related top-down suppression deficit in the early stages of cortical visual memory processing. Proc. Natl. Acad. Sci. U.S.A. 105, 13122–13126. 10.1073/pnas.0806074105 18765818 PMC2529045

[B19] Gazzaley A. Cooney J. W. McEvoy K. Knight R. T. D'Esposito M. (2005a). Top-down enhancement and suppression of the magnitude and speed of neural activity. J. Cogn. Neurosci. 17, 505–517. 10.1162/0898929053279522 15814009

[B20] Gazzaley A. Cooney J. W. Rissman J. D'Esposito M. (2005b). Top-down suppression deficit underlies working memory impairment in normal aging. Nat. Neurosci. 8, 1298–1300. 10.1038/nn1543 16158065

[B21] Georgiou-Karistianis N. Tang J. Mehmedbegovic F. Farrow M. Bradshaw J. Sheppard D. (2006). Age-related differences in cognitive function using a global local hierarchical paradigm. Brain Res. 1124, 86–95. 10.1016/j.brainres.2006.09.070 17069772

[B22] Germine L. T. Duchaine B. Nakayama K. (2011). Where cognitive development and aging meet: face learning ability peaks after age 30. Cognition 118, 201–210. 10.1016/j.cognition.2010.11.002 21130422

[B23] Goldman-Rakic P. S. (1995). Cellular basis of working memory. Neuron 14, 477–485. 10.1016/0896-6273(95)90304-6 7695894

[B24] Greenwood P. M. Parasuraman R. (1999). Scale of attentional focus in visual search. Percept. Psychophys. 61, 837–859. 10.3758/BF03206901 10498999

[B25] Herzog M. Fahle M. (1997). The role of feedback in learning a vernier discrimination task. Vis. Res. 37, 2133–2141. 10.1016/S0042-6989(97)00043-6 9327060

[B26] Herzog M. Fahle M. (1999). Effects of biased feedback on learning and deciding in a vernier discrimination task. Vision Res. 39, 4232–4243. 10.1016/S0042-6989(99)00138-8 10755160

[B27] Hildebrandt A. Wilhelm O. Schmiedek F. Herzmann G. Sommer W. (2011). On the specificity of face cognition compared with general cognitive functioning across adult age. Psychol. Aging 26, 701–715. 10.1037/a0023056 21480718

[B28] Hildebrandt A. Sommer W. Herzmann G. Wilhelm O. (2010). Structural invariance and age-related performance differences in face cognition. Psychol. Aging 25, 794–810. 10.1037/a0019774 20822255

[B29] Konar Y. Bennett P. J. Sekuler A. B. (2013). Effects of aging on face identification and holistic face processing. Vision Res. 88, 38–46. 10.1016/j.visres.2013.06.003 23806271

[B30] Leder H. Candrian G. Huber O. Bruce V. (2001). Configural features in the context of upright and inverted faces. Perception 30, 73–83. 10.1068/p2911 11257979

[B31] Lee Y. Smith C. R. Grady C. L. Hoang N. Moscovitch M. (2014). Broadly tuned face representation in older adults assessed by categorical perception. J. Exp. Psychol. Hum. Percept. Perform. 40, 1060–1071. 10.1037/a0035710 24490946

[B32] Lincourt A. E. Folk C. L. Hoyer W. (1997). Effects of aging on voluntary and involuntary shifts of attention. Aging Neuropsychol. Cogn. C4, 290–303. 10.1080/13825589708256654 29053089

[B33] Lott L. A. Haegerstrom-Portnoy G. Schneck M. E. Brabyn J. A. (2005). Face recognition in the elderly. Optom. Vis. Sci. 82, 874–881. 10.1097/01.opx.0000180764.68737.91 16276318

[B34] Meinhardt-Injac B. Persike M. Meinhardt G. (2011). The context effect in face matching: effects of feedback. Vision Res. 51, 2121–2131. 10.1016/j.visres.2011.08.004 21854801

[B35] Meinhardt-Injac B. Persike M. Meinhardt G. (2014a). Development of visual systems for faces and objects: further evidence for prolonged development of the face system. PLoS ONE. 9:e99942. 10.1371/journal.pone.0099942 24955959 PMC4067275

[B36] Meinhardt-Injac B. Persike M. Meinhardt G. (2014b). Holistic processing and reliance on global viewing strategies in older adults' face perception. Acta Psychol. 151, 155–163. 10.1016/j.actpsy.2014.06.001 24977938

[B37] Murray J. E. Halberstadt J. Ruffman T. (2010). The face of aging: sensitivity to facial feature relations changes with age. Psychol. Aging 25, 846–850. 10.1037/a0019864 20677879

[B38] Navon D. (1977). Forest before trees: the precedence of global features in visual perception. Cogn. Psychol. 9, 353–383. 10.1016/0010-0285(77)90012-3

[B39] Obermeyer S. Kolling T. Schaich A. Knopf M. (2012). Differences between old and young adults' ability to recognize human faces underlie processing of horizontal information. Front. Aging Neurosci. 4:3. 10.3389/fnagi.2012.00003 22536184 PMC3332157

[B40] Pfutze E. M. Sommer W. Schweinberger S. R. (2002). Age-related slowing in face and name recognition: evidence from event-related brain potentials. Psychol. Aging 17, 140–160. 10.1037/0882-7974.17.1.140 11931282

[B41] Prakash R. S. Erickson K. I. Colcombe S. J. Kim J. S. Voss M. W. Kramer A. F. (2009). Age-related differences in the involvement of the prefrontal cortex in attentional control. Brain Cogn. 71, 328–335. 10.1016/j.bandc.2009.07.005 19699019 PMC2783271

[B42] Quigley C. Andersena S. Schulze L. Grunwald M. Müller M. (2010). Feature-selective attention: evidence for a decline in old age. Neurosci. Lett. 474, 5–8. 10.1016/j.neulet.2010.02.053 20219631

[B43] Rajah M. N. D'Esposito M. (2005). Region-specific changes in prefrontal function with age: a review on pet and fmri studies on working and episodic memory. Brain 128, 1964–1983. 10.1093/brain/awh608 16049041

[B44] Richler J. J. Cheung O. S. Gauthier I. (2011). Holistic processing predicts face recognition. Psychol. Sci. 22, 464–471. 10.1177/0956797611401753 21393576 PMC3077885

[B45] Richler J. J. Gauthier I. (2013). When intuition fails to align with data: a reply to rossion. Vis. Cogn. 21, 1–13. 10.1080/13506285.2013.796035 24307858 PMC3845673

[B46] Richler J. J. Gauthier I. Wenger M. J. Palmeri T. L. (2008a). Holistic processing of faces: perceptual and decisional components. J. Exp. Psychol. Learn. Mem. Cogn. 34, 328–342. 10.1037/0278-7393.34.2.328 18315409

[B47] Richler J. J. Tanaka J. W. Brown D. D. Gauthier I. (2008b). Why does selective attention to parts fail in face processing? J. Exp. Psychol. Learn. Mem. Cogn. 34, 1356–1368. 10.1037/a0013080 18980400

[B48] Rossion B. (2013). The composite face illusion: a whole window into our understanding of holistic face perception. Vis. Cogn. 21, 139–253. 10.1080/13506285.2013.772929

[B49] Salthouse T. A. (1996). The processing-speed theory of adult age differences in cognition. Psychol. Rev. 103, 403–428. 10.1037/0033-295X.103.3.403 8759042

[B50] Salthouse T. A. (2000). Aging and measures of processing speed. Biol. Psychol. 54, 35–54. 10.1016/S0301-0511(00)00052-1 11035219

[B51] Schmitz T. Cheng F. De Rosa E. (2010). Failing to ignore: paradoxical neural effects of perceptual load on early attentional selection in normal aging. J. Neurosci. 30, 14750–14758. 10.1523/JNEUROSCI.2687-10.2010 21048134 PMC6633629

[B52] Searcy J. H. Bartlett J. C. Memon A. (1999). Age differences in accuracy and choosing in eyewitness identification and face recognition. Mem. Cogn. 27, 538–552. 10.3758/BF03211547 10355242

[B53] Sekular R. Sekular A. B. (2000). Visual perception and cognition, in Oxford Textbook of Geriatric Medicine, eds Evans J. G. Williams T. F. Michel J.-P. Beattie L. (Oxford: Oxford University Press), 874–880

[B54] Sekunova A. Barton J. J. S. (2008). The effects of face inversion on the perception of longrange and local spatial relations in eye and mouth configuration. J. Exp. Psychol. Hum. Percept. Perform. 34, 91129–91135. 10.1037/0096-1523.34.5.1129 18823200

[B55] Tanaka J. W. Farah M. J. (1993). Parts and wholes in face recognition. Q. J. Exp. Psychol. 45, 34–79. 8316637 10.1080/14640749308401045

[B56] Tanaka J. W. Sengco J. A. (1997). Features and their configuration in face recognition. Mem. Cogn. 25, 583–592. 10.3758/BF03211301 9337578

[B57] Vanneste S. Pouthas V. (1999). Timing in aging: the role of attention. Exp. Aging Res. 25, 49–67. 10.1080/036107399244138 11370109

[B58] West R. (1996). An application of prefrontal cortex function theory to cognitive aging. Psychol. Bull. 120, 272–292. 10.1037/0033-2909.120.2.272 8831298

[B59] Wilhelm O. Herzmann G. Kunina O. Danthiir V. Schacht A. Sommer W. (2010). Individual differences in perceiving and recognizing faces - one element of social cognition. J. Pers. Soc. Psychol. 90, 530–548. 10.1037/a0019972 20677889

[B60] Yin R. K. (1969). Looking at upside-down faces. J. Exp. Psychol. 81, 141–145. 10.1037/h0027474

[B61] Young A. M. Hellawell D. Hay D. C. (1987). Configural information in face perception. Perception 16, 747–759. 10.1068/p160747 3454432

