# Supplementary material for: Holistic face perception in young and older adults: effects of feedback and attentional demand
Source: Front Aging Neurosci. 2014 Oct 22;6:291. doi: 10.3389/fnagi.2014.00291 (PMC4208490; doi:10.3389/fnagi.2014.00291)
Supplement: Supplementary file 1 [file DataSheet1.PDF]

APPENDIX: RESULTS FOR SDT MEASURES

In order to enable comparison of the results of this study with results from studies using measures derived from signal detection theory (e.g. **Konar et al.** (2013)) we provide a results overview for d' and response criterion c as the measures for sensitivity and bias. Standard formulae were used, i.e. $d' = z(Hit) - z(FA)$ and $c = -(z(Hit) + z(FA))/2$. ANOVA of both measures and evaluation of effects yielded no qualitative differences compared to the results obtained with proportion correct, Pc, and the error proportion measure, Q.

Table 3. Results for the sensitivity estimate, d', and response criterion, c. Results for congruent (CC) and incongruent (IC) trials are listed in the same line.

Age group	Feedback	Cue position	Time	$d'(CC)$	$d'(IC)$	$c(CC)$	$c(IC)$	N
young	FB	1	50	2.91	2.10	-0.11	0.07	23
young	FB	1	233	3.03	2.62	0.01	0.02	23
young	FB	1	633	3.27	2.71	-0.02	-0.03	23
young	FB	2	50	2.69	1.74	-0.04	0.06	23
young	FB	2	233	2.79	1.92	-0.15	0.06	23
young	FB	2	633	2.83	1.93	-0.20	0.01	23
young	NoFB	1	50	3.13	1.98	0.06	0.34	23
young	NoFB	1	233	3.25	2.31	0.01	0.33	23
young	NoFB	1	633	3.30	2.50	0.08	0.26	23
young	NoFB	2	50	2.73	1.44	-0.04	0.44	23
young	NoFB	2	233	3.01	1.61	0.04	0.28	23
young	NoFB	2	633	3.11	1.56	-0.04	0.20	23
old	FB	1	50	2.05	1.17	-0.16	-0.31	20
old	FB	1	233	2.59	1.54	-0.22	-0.04	20
old	FB	1	633	2.89	2.00	-0.13	0.11	20
old	FB	2	50	1.38	0.49	-0.22	-0.12	20
old	FB	2	233	1.87	0.85	-0.28	0.00	20
old	FB	2	633	2.17	0.64	-0.23	-0.14	20
old	NoFB	1	50	2.27	0.86	-0.24	-0.17	20
old	NoFB	1	233	2.75	1.43	-0.14	0.03	20
old	NoFB	1	633	2.90	1.36	-0.08	0.18	20
old	NoFB	2	50	1.90	0.48	-0.14	-0.11	20
old	NoFB	2	233	2.54	0.73	-0.07	0.01	20
old	NoFB	2	633	2.77	0.94	-0.19	0.21	20
